# Age-Related Changes in EEG Signal Complexity and Behavioral Variability from Childhood to Adulthood: A Multiscale Entropy Approach

**DOI:** 10.3390/e28040390

**Published:** 2026-04-01

**Authors:** Brenda Y. Angulo-Ruiz, Vanesa Muñoz, Elena I. Rodríguez-Martínez, Carlos M. Gómez

**Affiliations:** Human Psychobiology Laboratory, Experimental Psychology Department, University of Seville, 41018 Seville, Spain; bangulo@us.es (B.Y.A.-R.); lmunnoz@us.es (V.M.); elisroma@us.es (E.I.R.-M.)

**Keywords:** electroencephalography, multiscale entropy, neural complexity, brain development, behavioral variability, age-related changes

## Abstract

The complexity of physiological signals provides insight into the maturation and functional organization of the developing brain. This study investigated age-related changes in electroencephalographic (EEG) signal complexity and their association with behavioral variability in 240 participants aged 6 to 29 years. EEG signals were recorded during the resting state, and Multiscale Entropy (MSE) was computed across 34 temporal scales, grouped into fine, medium, and coarse scales. Behavioral variability was assessed using measures from Oddball and Delayed Match-to-Sample tasks. Quadratic regression analyses characterized age-related changes in MSE across scalp regions, and Pearson correlations evaluated associations between age-adjusted residuals of MSE and behavioral variability. The results showed that MSE changed with age across temporal scales in all cortical regions. Developmentally, MSE showed a significant age-related increase at fine scales across the entire scalp, region-specific decreases at medium scales, and a generalized decrease at coarse scales. Behavioral variability decreased with age across both tasks. Notably, fine-scale age residual MSE in central and posterior regions was negatively correlated with the coefficient of variation in the Oddball task, indicating that higher neural complexity supports more stable performance. These findings suggest scale- and region-specific age-related changes in neural complexity and suggest that fine-scale MSE captures aspects of brain maturation related to behavioral stability beyond traditional variability measures.

## 1. Introduction

The complexity of biological systems manifests itself through dynamic fluctuations operating across multiple temporal and spatial scales [1]. In neuroscience and physiology, this complexity is reflected in time series such as the electroencephalogram (EEG), whose variability cannot be fully captured using traditional linear metrics. Measures that quantify the temporal structure of these fluctuations have therefore become increasingly important for understanding the organization of physiological systems. Among these approaches, Multiscale Entropy (MSE) has emerged as a powerful tool for characterizing the complexity and unpredictability of physiological signals across different temporal scales [2,3,4,5].

Conceptually, MSE integrates a coarse-graining procedure, which generates new time series at different temporal scales, with the computation of sample entropy at each scale. This framework allows the assessment of how signal regularity changes when patterns of varying durations are considered [1,3,6,7]. Using this approach, healthy physiological systems typically show greater complexity across a broad range of scales, whereas aging and various pathological conditions are associated with a reduction in complexity [2,8,9,10,11,12,13,14,15]. These findings support the view that physiological complexity reflects the adaptive capacity and functional integration of biological systems.

Within the developmental context, MSE has emerged as a sensitive indicator of brain maturation. EEG studies in children and adolescents have reported age-related increases in resting-state signal complexity across multiple temporal scales, with especially pronounced changes in frontocentral regions [7,16]. Longitudinal research in infants, including preterm babies, has similarly shown increases in complexity and regional changes in functional connectivity during the first two years of life, suggesting that MSE captures processes of the progressive organization and specialization of cortical networks [17,18]. In contrast, analyses in adult and older populations have reported reductions in complexity in conditions such as mild cognitive impairment and Alzheimer’s disease, as well as entropy modulations associated with affective symptoms [5,13,19]. Together, this body of work highlights the potential of MSE as a biomarker of functional brain integrity and neuronal adaptation across the lifespan.

Beyond developmental and clinical contexts, recent studies have jointly examined measures of neural variability (standard deviation—SD—and sample entropy) and behavioral variability (reaction time—RT SD) in sustained attention tasks, revealing distinct topographic patterns of correlation between each neural index and performance variability [20,21]. In perceptual paradigms, greater irregularity in the spatial patterns of brain activity has been associated with lower RT SD, supporting the idea that greater organized variability or complexity may promote more stable performance [21]. These findings support the idea that combining MSE with classical behavioral metrics, such as the SD and coefficient of variation (CV) of RT, may provide a more comprehensive characterization of the relationship between neural dynamics and behavior.

Despite these advances, relatively few studies have examined how neural complexity during the resting state relates to behavioral variability across development. In this sense, the analysis of MSE in a large sample of individuals aged 6 to 29 years provides an opportunity to characterize in detail how the complexity of physiological signals evolves across critical developmental stages, from late childhood and adolescence to young adulthood. Building on the theoretical framework of physiological complexity and prior evidence on MSE and neurophysiological development, the present study aims to describe age-dependent changes in the temporal structure of the recorded signals and to explore their implications for understanding processes of maturation and functional organization during development.

The present study addresses this question by examining the MSE metric across late childhood, adolescence, and young adulthood and analyzes the association between MSE and behavioral performance in two cognitive tasks (Oddball and the Delayed Match-to-Sample test—DMTS), comparing the added value of MSE during the resting state when analyzed vs. traditional indices of RT variability, such as SD and CV. This approach allows us to assess whether the temporal complexity of physiological signals provides specific information about performance stability in attentional (Oddball) and working memory processes (DMTS).

Based on previous theoretical and empirical work, we hypothesize that fine-scale MSE will increase with age, reflecting the progressive maturation of local cortical networks, while medium- and coarse-scale MSE will show region-specific or global decreases, indicating the developmental refinement of long-range neural interactions. Furthermore, we predict that higher resting-state MSE, particularly at fine scales, will be associated with lower behavioral variability (SD and CV) in attentional and working memory tasks, supporting the idea that neural complexity contributes to more stable cognitive performance. Finally, we expect that MSE will provide complementary information to traditional metrics of EEG and behavioral variability, capturing aspects of functional brain organization and maturation that are not fully reflected by conventional metrics [20,21,22,23].

## 2. Materials and Methods

### 2.1. Sample

A total of 240 subjects (116 women) aged between 6 and 29 years (M = 14.85, SD = 5.99) were recruited. The sample data included in the present study have been previously analyzed for different research purposes and for different signal analyses [8,10,24]. No significant differences in age were found between men and women in the extracted subsample, t(238) = 0.024, *p* = 0.98. No participants reported a history of neurological or psychological illness or cognitive impairment. Participants over the age of 18 were university students with normal academic performance, while the younger participants were primary and secondary school students from schools in the metropolitan area of Seville.

This study was conducted in accordance with the principles of the Declaration of Helsinki, and all participants provided written informed consent. The experimental protocol was approved by the Biomedical Research Ethics Committee of the Autonomous Community of Andalusia.

### 2.2. EEG Recording

Spontaneous electrical activity was recorded for 3 min in an eye-open condition. Participants were instructed to fix their gaze on a centralized white cross (2 × 2 cm) against a black background to minimize ocular artifacts and eye movements. They were asked to blink as little as possible and remain in a comfortable position. The recording was performed using 32 electrodes mounted on an electrode cap (ELECTROCAP) in accordance with the international 10–20 system (Fp1, Fpz, Fp2, F7, F3, Fz, F4, F8, FC5, FC1, FC2, FC6, M1, T7, C3, Cz, C4, T8, M2, CP5, CP1, CP2, CP6, P7, P3, Pz, P4, P8, Poz, O1, Oz, O2). For eye movement recording, two electrodes were placed on the outer edge of each eye to detect horizontal movements, and two electrodes were placed above and below the left eye for vertical movements. For signal analysis, the eye and mastoid electrodes were removed for a total of 30 electrodes analyzed. Impedance was kept below 10 KΩ. Data were acquired in direct current at 512 Hz, with an amplification gain of 20,000, using an analog–digital acquisition and analysis system (ANT Neuro b.v. Amplifiers, (Welbergwen 74, 7556PE Hengelo, The Netherlands)). No filtering was applied during recording.

### 2.3. Data Analysis

#### 2.3.1. EEG Pre-Processing

The data were analyzed in Matlab R2022b using the EEGLAB software package (version 2021.0) [25]. Signal preprocessing consisted of applying a 47–53 Hz notch filter (*eegfiltnew*), an average reference, and an artifact subspace reconstruction algorithm (*pop_clean_rawdata*) [26]. Subsequently, independent component analysis (ICA) was performed to identify and remove artifacts associated with blinking, muscle activity, and other movements, using the *pop_runica* function with the natural gradient algorithm [27,28]. The classification of independent components was performed using the ICLabel extension, and rejections were made following the guidelines proposed by Pion-Tonachini et al. [29].

To assess possible age-related biases in data quality after preprocessing, the sample was divided into two groups of 116 participants each (6–13 years and 13–29 years). The groups were then compared in terms of the number of accepted epochs and the number of independent components retained after ICA. The number of accepted epochs differed between groups (t(117.38) = −3.22, *p* = 0.002), group 1 (M = 88.36; SD = 5.34) and group 2 (M = 89.97, SD = 0.54), with a small effect size (d = −0.42). Likewise, the number of independent components accepted showed a statistically significant difference (t(229.98) = 5.10, *p* < 0.001), with means of (group 1: (M = 21.82, SD = 0.57) and group 2: (M = 21.44, SD = 0.56)) and a moderate effect size (d = 0.67). In both cases, participants retained on average more than 21 of the 30 possible components (≈70%). These comparisons were performed to verify that the signal amount and dimensionality of the data after preprocessing were comparable between groups. Although the differences were statistically significant, their magnitude was minimal, indicating comparable datasets. After the reconstruction of the EEG signal, 2 s epochs in which the amplitude exceeded ±120 µV in any electrode (*eegthresh*) were automatically rejected.

#### 2.3.2. Multiscale Entropy

Signal complexity was estimated using MSE, which was computed for all participants and electrodes using the Multiscale Sample Entropy function in MATLAB (version 2022b) [30], based on the approach proposed by Costa et al. [1]. This method allows the dynamics of the signal to be characterized by calculating the sample entropy (*SE*) at multiple time scales, applying a coarse-graining procedure that generates progressively smoother representations of the original time series (length τ). At each scale, the signal was obtained by averaging consecutive points (*p*) of the original time series, and the sample entropy was estimated by comparing the recurrence of patterns of length “*m*” with that of patterns of length “*m* + 1” in non-overlapping windows of different sizes. The criterion for similarity between patterns was defined by a tolerance threshold (*r*), normalized to the SD of the EEG signal, according to the relationship k < *r* < SD. The following equation (Equation (1)) was used to calculate MSE [30]:

(1) $$SE=log\;\frac{p^{m}\left(r\right)}{p^{\left(m+1\right)}\left(r\right)}$$

Following the recommendations of previous studies on EEG signal complexity [31,32,33,34,35], the parameters were set at *m* = 2 and *r* = 0.5. MSE was estimated considering time scales between 1 and 34, which allowed the complexity of the signal to be evaluated from fine to coarse time scales. This approach also enabled an indirect analysis of low-frequency components (≤7.53 Hz). The maximum time scale corresponded to a temporal resolution of 66.4 milliseconds (ms) per point and a total of 30 time points per trial. A detailed description of the number of points, sampling periods, and frequencies associated with each MSE scale is presented in Supplementary Table S1.

From an interpretative perspective, higher MSE values indicate greater signal irregularity, as patterns of length “*m* + 1” are repeated less frequently than those of length “*m*”. This reflects more complex, flexible, and less structured brain activity, characteristics commonly associated with healthy biological systems [18]. Conversely, low MSE values indicate greater regularity or predictability in signal dynamics, which can be interpreted as a decrease in its informational content [32,36].

Recent work has linked the coarse-grained procedure of the MSE to spectral power analysis using Haar wave-based approximations [37]. From this perspective, the fine time scales of MSE (shorter time windows) incorporate information from the entire EEG frequency spectrum, while longer time scales mainly reflect low-frequency components [37]. Thus, MSE does not simply measure randomness, but rather the structural complexity of the system at different organizational levels [1,3]. Fine scales are associated with information processing mechanisms in local cortical networks, where an increase in entropy reflects the functional maturation of these circuits. On the other hand, coarse scales act as a filter that captures long-range integration between distant regions [38]. Thus, changes at these scales would allow us to track the refinement of global connectivity during development, distinguishing between stochastic noise and real biological complexity.

### 2.4. Statistical Analysis

#### 2.4.1. Resting-State EEG Analysis

Statistical analyses were performed using SPSS (version 25) and MATLAB (version 2022b). For the processing of electroencephalographic data, a dimensionality reduction strategy was applied based on the clustering of electrodes into nine topographic regions covering the entire scalp: left anterior (Fp1, F7, F3), left central (FC5, T7, C3, CP5), left posterior (P7, P3, O1), middle anterior (Fpz, Fz), middle central (FC1, FC2, Cz, CP1, CP2), middle posterior (Pz, POz, Oz), right anterior (Fp2, F4, F8), right central (FC6, C4, T8, CP6), and right posterior (P4, P8, O2).

In addition, the MSE scales were grouped into three ranges, following an approach described previously [39]. Thus, the 34 original scales were reduced to a smaller number of dimensions: (i) fine scales, ranging from scale 1 (1.95 ms; 1024 time points) to scale 13 (25.39 ms; 78 time points); (ii) medium scales, ranging from scale 14 (27.34 ms; 73 time points) to 23 (44.92 ms; 44 time points); and coarse scales, ranging from scale 24 (46.88 ms; 42 time points) to 34 (66.4 ms; 30 time points). A list of the different scales used appears in Supplementary Table S1.

For statistical analysis, curvilinear estimates were calculated using linear, inverse, exponential, and quadratic regression models to evaluate the relationship between MSE and the age of participants. Comparison between models indicated that quadratic regressions provided the best fit to the data, so only these results are reported in the present study. The progression of MSE values over time was examined across the different regions of the scalp as defined above. Subsequently, quadratic regression analyses were performed between age (expressed in days) and each of the MSE scale ranges (fine, medium, and coarse).

To determine whether the results depended on the categorization of temporal scales into fine, medium, and coarse, an additional analysis was performed, evaluating the relationship between age and MSE across all scales. This analysis allowed us to examine the robustness of the findings with respect to the grouping of temporal scales. First, global MSE values were calculated for each participant by averaging the electrodes within three ranges of temporal scales: fine (1–13), medium (14–23), and coarse (24–34). For each scale range, simple linear regression models were then fitted to examine the relationship between age (in days) and global MSE values. Second, the relationship between age and MSE was evaluated at the regional level across the 34 individual temporal scales. The electrodes were grouped into nine defined cortical areas. For each area and scale, Pearson correlations were calculated between age and the mean MSE value for the area.

#### 2.4.2. Behavioral Analysis

Given that previous studies have reported an association between MSE and behavioral variability [20,21], an additional analysis was conducted to further examine this relationship. For this purpose, RTs data from two cognitive tasks (Oddball and DMTS) were analyzed using a previously published dataset [40]. The RTs tasks were recorded at a different time point within the same experimental protocol on the same day. The experimental procedure followed for each task was as follows:

Delayed Match-to-Sample (DMTS) task: Participants completed 128 trials organized in four blocks. Each trial began with the presentation of a sample stimulus (S1) for 1000 ms, followed by a 1500 ms delay. Two comparison stimuli (S2)—one identical to S1 and one novel—were then displayed for 2000 ms on the left and right sides of the screen. Participants indicated which S2 matched S1 by pressing the corresponding button. Auditory feedback signaled correct or incorrect responses. Stimuli were cartoon images, balanced for left and right presentation, and randomized for each participant. The SD and CV of RTs were calculated for each participant.

Oddball task: Participants completed 120 trials in a single block, with 25% novel and 75% repeated cartoon stimuli. Each stimulus was presented for 700 ms, followed by a 700 ms inter-stimulus interval. Participants responded only to novel stimuli. For this task, RTs, SDs, and CVs were also computed.

#### 2.4.3. MSE vs. Behavioral Correlations

To properly characterize the influence of age on behavioral measures, various models (linear, inverse, and exponential) were evaluated, with the quadratic model providing the best fit to the data. Therefore, all variables were adjusted for age (in days) using this model. Consequently, to examine the relationship between resting-state MSE and subsequent behavioral performance, the residuals of the behavioral variability measures (SD and CV) obtained in the Oddball and DMTS tasks were used, as well as the residuals of the MSE measures on fine-scale regional levels, all adjusted for age in days.

Thus, the MSE–behavior correlations are based exclusively on variability not explained by age, avoiding potential confounding effects associated with development. Finally, Pearson correlations were calculated between the residuals of the neurophysiological measures and those of the behavioral variables.

All results are reported, applying multiple comparison correction using the False Discovery Rate (FDR) procedure [41]; additionally, missing values (NaN) were excluded from the analyses, and outliers were removed when values exceeded ±3 standard deviations.

## 3. Results

**Figure 1:**
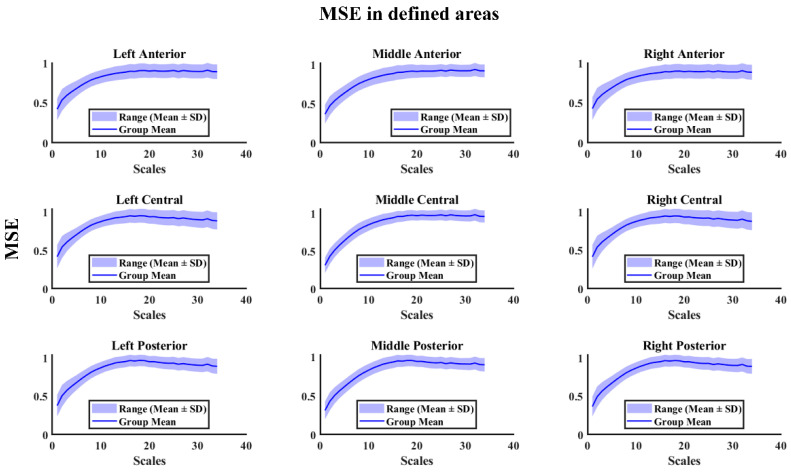
Multiscale Entropy (MSE) across temporal scales in predefined cortical regions. Mean MSE values (solid line) and variability range (shaded area representing mean ± standard deviation (SD) across participants) are shown as a function of scale for anterior, central, and posterior regions in the left, middle, and right areas.

Figure 1 shows the Multiscale Entropy values according to scale in the different regions considered. An increase in MSE is observed as the time scale increases in all the areas analyzed.

**Figure 2:**
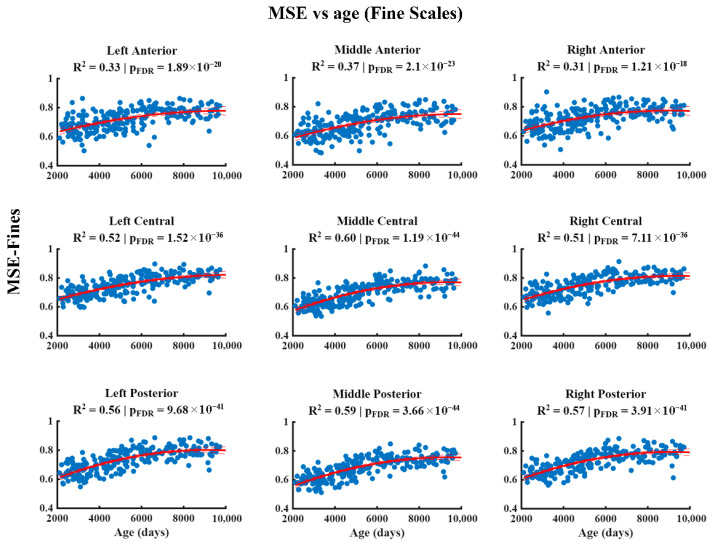
The relationship between Multiscale Entropy (MSE) and age (in days) at fine scales. The figure displays the quadratic regression model (red line) that describes the positive association between age (*x*-axis) and MSE (*y*-axis) in the anterior, central, and posterior regions in the left, middle, and right hemispheres. *p*-values have been corrected for multiple comparisons using the False Discovery Rate (FDR) method.

**Figure 3:**
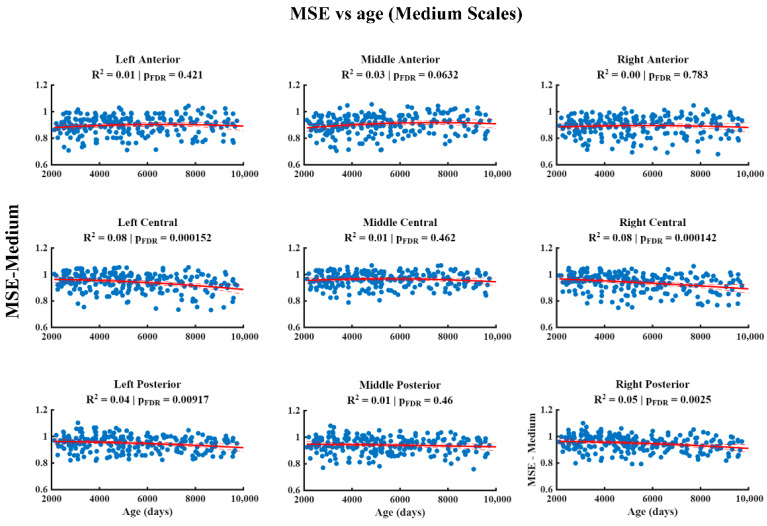
The relationship between Multiscale Entropy (MSE) and age (in days) at medium scales. The figure displays the quadratic regression model (red line) describing the significant negative association between age (*x*-axis) and MSE (*y*-axis) in the left central, right central, left posterior and right posterior regions after multiple comparisons using the False Discovery Rate (FDR) method.

**Figure 4:**
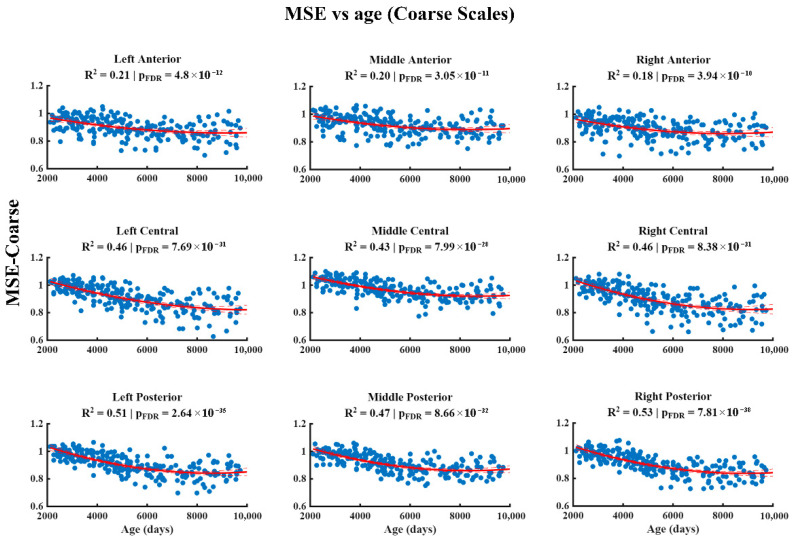
The relationship between Multiscale Entropy (MSE) and age (in days) at coarse scales. The figure displays the quadratic regression model (red line) describing the negative associations between age (*x*-axis) and MSE (*y*-axis) in the anterior, central, and posterior regions of the left, middle, and right hemispheres. *p*-values have been corrected for multiple comparisons using the False Discovery Rate (FDR) method.

The results of the relationship between MSE and age at different temporal scales are shown in Figure 2, Figure 3 and Figure 4. At the fine scale (Figure 2), a significant positive association between MSE and age is observed across all regions of the scalp, characterized by a quadratic pattern that remains significant after correction for multiple comparisons (FDR). At the medium scale (Figure 3), the significant negative associations corrected by FDR are restricted to the left central, right central, left posterior, and right posterior regions, whilst the remaining areas do not show robust effects. Finally, at the coarse scale (Figure 4), significant negative associations between MSE and age are observed in all the regions analyzed, which were also characterized by a quadratic pattern and remained significant after FDR correction.

**Figure 5:**
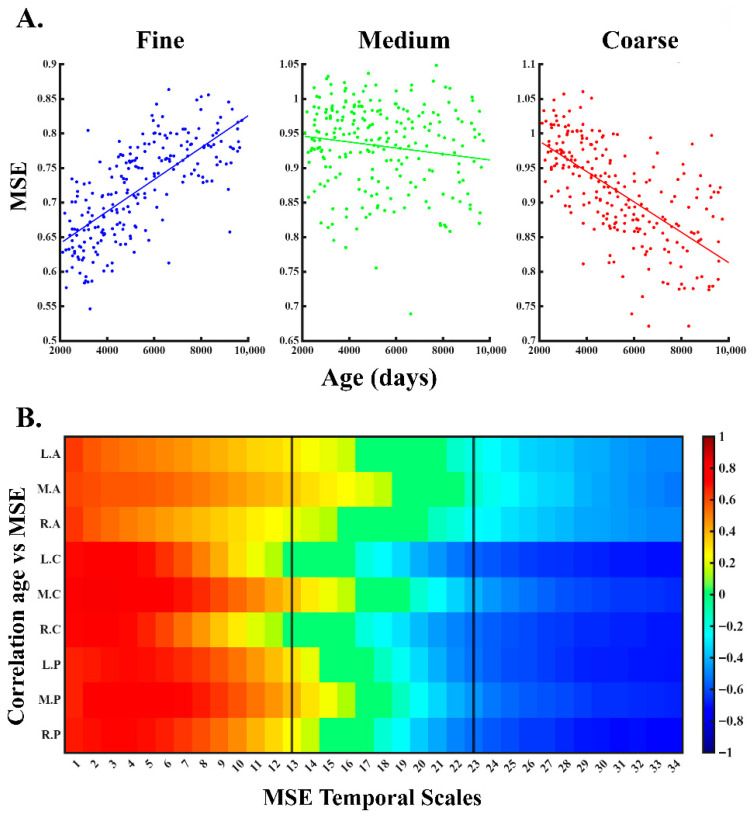
The relationship between age and MSE across different temporal scales and electrodes. (**A**) The fitting of a linear regression model between age and global (averaging across electrodes) MSE for fine, medium, and coarse scales. (**B**) Age–MSE Pearson correlations scale by scale and by cortical areas (FDR–corrected). The black vertical lines in the middle of the figure indicate the boundaries used to define the fine, medium, and coarse scales (code for brain areas: left (L), medial (M), right (R), anterior (A), central (C), posterior (P)).

To assess whether the results depended on the categorization of the temporal scales, we performed a complementary analysis examining the relationship between age and MSE both at the global level (Figure 5A) and scale by scale (Figure 5B). The results show that the previously selected range for fine, medium, and coarse scales coincides with the trend changes in the relationship between MSE and age across the whole scalp.

**Figure 6:**
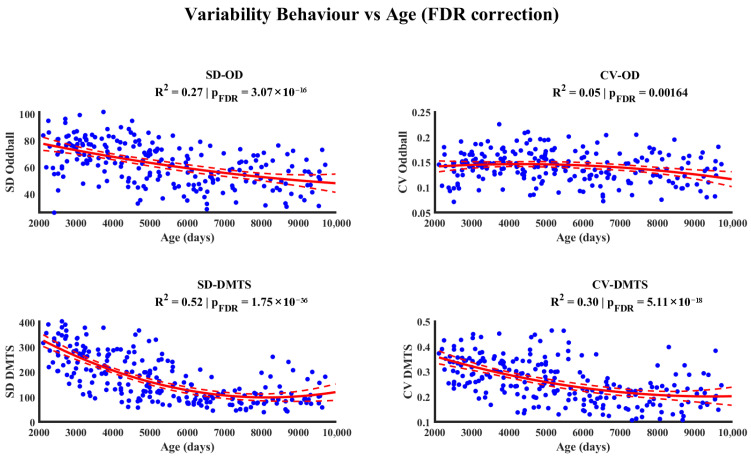
The relationship between age and behavioral variability. A quadratic regression model (red line) is fitted to describe the relationship between age (*x*-axis) and the behavioral variability measures, standard deviation (SD), and coefficient of variation (CV), obtained from the Oddball and DMTS tasks. *p*-values have been corrected for multiple comparisons using the False Discovery Rate (FDR) method. (OD: Oddball task; DMTS: Delayed Match to Sample).

Figure 6 presents the results of the relationship between age and behavioral variability measures (standard deviation (SD) and coefficient of variation (CV)) from the Oddball and DMTS tasks. The quadratic regression models show significant associations after correction for multiple comparisons (FDR). The analyses indicate that the variability in behavioral performance changes throughout development, with a general tendency to decrease with age.

Finally, to evaluate the relationship between neural complexity and variability in RT responses (SD and CV) in the Oddball and DMTS tasks, Pearson correlations were calculated using the residuals of MSE measures at fine scales by region and the residuals of behavioral measures, both adjusted for age using a quadratic model. After correction for multiple comparisons (FDR), significant negative correlations were observed only between the CV in the Oddball task and fine-scale MSE in the left central, right central, and right posterior regions. These results are reported in Table 1. Correlations between the age residual measures at medium and coarse scales and the behavioral measures did not show a significant correlation after FDR correction.

## 4. Discussion

### 4.1. Age-Related Changes in MSE and Brain Maturation

The present results show a monotonic increase in MSE with the scale order when the entire sample was averaged across ages. The developmental trajectory of MSE at fine scales increased with age and was best fitted by a quadratic model compared to other tested models (linear, inverse, and exponential), with only a small decrease observed in the oldest participants. In contrast, MSE at medium scales showed age dependencies limited to some regions. At coarse scales, MSE decreased with age and was also best described by a quadratic function.

A similar developmental pattern was observed in behavioral measures. RT variability, indexed by the SD and CV of the Oddball and DTMTS tasks, decreased with age and followed a quadratic trajectory. Given that most variables exhibited quadratic relationships with age, age-related effects were removed by computing residuals for all measures. This approach allowed us to examine whether individual differences in MSE were associated with behavioral variability once age effects were eliminated. Using this approach, significant associations were observed in specific brain regions at fine temporal scales, where MSE residuals were related to the reaction time CV in the Oddball task.

The age-related pattern of MSE observed in the present study is consistent with previous developmental findings. Van Noordt and Willoughby, as well as McIntosh et al. [16,32], reported age-related increases in fine temporal scales within a similar age range, together with reduced age dependency at medium scales, which agrees with the present findings. Likewise, studies that have examined age-related changes at coarse scales during resting-state EEG [7,39] similarly reported age-related increases in MSE at fine scales, no significant changes at medium scales, and an inverse relationship with age at coarse scales. Our results further suggest that these changes are best described by quadratic functions, indicating rapid change during childhood, characterized by increases in MSE at fine scales, followed by relative stabilization during adolescence and a trend reversal in early adulthood.

Interestingly, this pattern could possibly be related to widely described processes of brain maturation, such as synaptic pruning, which reduces redundant connections, and the progressive reorganization of connections between cortical regions, facilitated by myelination and the maturation of white matter tracts, resulting in faster and more efficient communication [42]. Likewise, the MSE quadratic relationship with age resembles the maturational quadratic patterns observed in the development of cortical thickness across much of the human cortex [43].

This pattern of increases at fine scales and decreases at coarse scales further reflects the well-established developmental shift in EEG spectral power, characterized by age-related increases in the relative power of higher-frequency activity and decreases in lower-frequency activity [24]. Recent studies have linked the coarse-graining procedure of MSE to spectral power analysis [37], showing that fine scales capture rapid temporal fluctuations, whereas coarse scales largely overlap with low-frequency EEG components, such as delta and theta. From this perspective, and although these processes were not directly measured in the present study, both EEG power [44] and complexity measures [45,46] may reflect brain connectivity. According to Chenxi et al. [47], low-frequency bands are associated with global brain activity, whereas high-frequency bands reflect local connectivity. Similarly, Wang et al. [48] demonstrated that multiscale complexity is not uniform: different scales reflect distinct dimensions of functional architecture, such that entropy at slow scales is associated with long-range connectivity patterns, while faster scales are linked to local dynamics.

In this context, the increase in fine scales with age and decrease in coarse scales with age imply a progressive refinement of brain connectivity, characterized by enhanced local communication and a reduction in long-range functional connections. However, this decrease does not necessarily imply reduced functional integration. Rather, it may possibly reflect a process of refinement of neural connectivity associated with synaptic pruning and progressive myelination, promoting more effective and efficient communication between distant brain regions. These developmental changes are potentially consistent with the transition toward a more efficient “small-world” network organization, characterized by an optimal balance between local specialization and global integration [49,50,51,52].

### 4.2. Multiscale Functional Interpretation and Connectivity Organization

This network organization is not static; recent literature highlights that functional stability and recovery depend on dynamic hemispheric interactions, as well as on contextual factors such as the social environment and peer influence [53]. In the context of brain reorganization, the ability to transition between states of integration and segregation has been associated with the activation of compensatory pathways, such as the strengthening of connections in the contralesional hemisphere, suggesting that network complexity is a critical substrate for neurological resilience [54]. Moreover, this capacity may be facilitated or constrained by external influences, such that a supportive social environment could exert a protective effect on development and associated maturational processes [53]. From this broader perspective, segregation can be viewed as a marker of both maturity and plasticity, facilitating specialization and functional efficiency, the expression of which could possibly be influenced by a positive social environment.

Regarding MSE at medium temporal scales, our results revealed significant age-related changes, predominantly localized in central and posterior regions after FDR correction. This regional specificity suggests that dynamics at these scales may reflect functional reorganization processes characteristic of intermediate stages of brain maturation, especially in areas involved in sensory integration and higher-order visual processing [55]. From a multiscale perspective, whereas fine MSE scales may reflect local activity within microcircuits and coarse scales are linked to long-distance functional interactions and the integration of distributed networks [44,45], medium scales may represent a mesoscopic level associated with the coordination between anatomically and functionally related regions. Consequently, medium scales may act as a functional transition zone between these two organizational levels. The very low correlation between medium MSE scales and age (Figure 5B) suggests that these mesoscopic networks may mature earlier than those associated with fine and coarse scales, indicating the relatively early maturation of mesoscopic brain networks. However, the effects observed in the present study were spatially specific, pointing to a region-dependent trajectory of neural development, consistent with the heterochronic nature of human brain development, in which different cortical regions mature at different rates [56]. Nevertheless, the existence of a causal relationship between anatomical maturation, the dynamics of interhemispheric interactions, and MSE remains an open question for future research.

### 4.3. Relationship Between MSE and Behavioral Variability

Beyond developmental effects, the relationship between neural complexity and behavioral variability provides further insight into the functional relevance of MSE. Previous studies have shown that MSE values at fine temporal scales increase with age, whereas variability in reaction times (CV) during a concurrent face recognition task decreases [21]. This has been interpreted as evidence that greater neural variability supports enhanced information processing capacity, which in turn allows for more stable behavioral responses. However, when age was controlled for, the relationship between fine-scale MSE and RT variability was no longer reliable, suggesting, according to the authors, that the initial association primarily reflected maturational processes. Our findings in the Oddball task support this conclusion: both SD and CV decreased with age, indicating a close association with the age-related increase in the developmental trajectory of fine-scale MSE.

However, our main interest lies in individual differences after controlling for age. We were able to examine associations independent of developmental effects by removing the quadratic maturational contribution of both MSE and behavioral variability measures. Our results show a significant, although weakened, correlation at fine scales with CV, replicating the findings of McIntosh et al. [32]. In contrast, the relationship between CV and MSE at coarse scales, which was not examined in McIntosh et al. [32], showed a parallel decrease with age but disappeared once age was controlled for, indicating that this association was entirely driven by maturation. Our findings therefore suggest that increased MSE at fine scales, both across maturation and as an individual-difference characteristic, may reflect more specialized processing within brain modules, thereby promoting greater stability in RTs.

Resting-state MSE has been associated with general cognitive abilities. For example, Menon and Krishnamurthy [57] demonstrated that neural complexity estimated from resting-state fMRI (Functional Magnetic Resonance Imaging) predicts interindividual differences in fluid intelligence, a construct closely linked to working memory and reasoning processes. From this perspective, one might expect greater neural complexity to be associated with better performance in tasks requiring active information maintenance, such as the DMTS task [58,59]. However, our results show a significant relationship in the Oddball task, but not in the DMTS task, after controlling for age. Specifically, a negative correlation was observed between MSE and reaction time stability (CV), primarily in sensorimotor areas (left central, right central, and right posterior), which are functionally involved in response preparation and execution, and in attentional orientation and alerting networks [60,61].

One possible explanation is that neural complexity at fine scales reflects the dynamic flexibility of sensorimotor and attentional circuits, facilitating the rapid integration of incoming information and more efficient response preparation. This interpretation is particularly consistent in tasks involving continuous vigilance and the detection of rare events, such as the Oddball paradigm [13,22,62]. In contrast, the DMTS task depends mainly on the stability of representations maintained during the delay interval and on frontoparietal working memory mechanisms [57,58], as well as frontosubcortical control processes related to the selection of relevant information and the inhibition of distractors [63]. In this sense, our results suggest that these mechanisms may not be directly indexed by the fine-scale MSE measured in the central and posterior regions where the Oddball-related associations were observed.

Consistent with this interpretation, previous studies have shown that greater entropy or neural variability in sensorimotor and attentional networks is associated with more stable and less variable performance in attentional tasks [20,22,64]. Our results refine this relationship by showing that it is not uniformly distributed at the cortical level but is specifically localized in bilateral central regions and the right posterior region. The involvement of central areas suggests that local microdynamics in the sensorimotor cortex constitute a critical determinant of response accuracy in the motor execution required by the Oddball paradigm, in line with studies linking signal complexity/variability in somatomotor networks to performance stability [20,22,65].

On the other hand, the lateralization observed in the present study in posterior regions, with significant effects only in the right hemisphere, is consistent with the functional specialization of this hemisphere in attentional orientation and alerting networks [66,67]. In particular, the detection of rare stimuli in Oddball tasks preferentially recruits right parietotemporal regions and the ventral attention network, which are involved in the detection of salient changes and attentional ‘resetting’ [66,67]. Thus, while the left hemisphere is more strongly associated with motor output, the right hemisphere plays a dominant role in the detection of rare Oddball stimuli and in maintaining the alert state [66,67,68]. In this context, signal complexity at these fine scales in the right hemisphere may constitute a marker of the system’s efficiency in monitoring the environment and responding consistently to novelty, in line with studies linking complexity/entropy to more efficient attentional control and reduced performance variability [20,22,69]. Likewise, this complexity reflects how neuronal systems adapt to environmental demands, such that environmental factors can facilitate or limit behavioral efficiency through their influence on the involved circuits, exerting a potential protective effect when they are favorable [53].

### 4.4. Conclusions

Overall, the present results of the present study suggest that MSE represents a meaningful parameter for indexing EEG maturation, capturing functionally relevant changes in the dynamic organization of brain activity across development. In particular, the results at fine scales indicate that increased variability at fine scales is also an index of individual development, possibly reflecting the increased segregation of local modules, thereby promoting more efficient communication and enhancing the reliability of behavioral responses.

### 4.5. Limitations

This study has some limitations that should be taken into account when interpreting the results. First, its cross-sectional design means that the results reflect age-related differences at the population level rather than intra-individual changes over time; therefore, the observed trends should be interpreted as estimates of developmental trajectories. Furthermore, the correlational nature and the different time moments for recording EEG and behavioral responses preclude establishing direct causal relationships between neural complexity and behavioral variability. Although EEG and behavioral measures were recorded within the same experimental protocol, the present study focused on trait-like associations between individual differences in resting-state signal complexity and subsequent task performance. Consequently, while our findings indicate that higher fine-scale MSE relates to more stable performance, they should be interpreted as functional couplings rather than direct causality. Future research employing task-based EEG or experimental manipulations will be necessary to determine the precise causal dynamics between real-time neural complexity and behavioral stability.

Another limitation of this study is that the associations observed between resting-state signal complexity (MSE) and behavioral variability, age-corrected, were relatively small in magnitude (r ≈ −0.16 to −0.19). However, this range of correlations is common in studies examining the relationships between individual neurophysiological measures and behavior, where a single neural metric typically explains only a small proportion of the behavioral variance, as these are complex phenomena that depend on multiple factors such as genetics and environment [70,71]. In our case, the observed R^2^ corresponds to approximately 2.5% to 3.6% of explained variance. Furthermore, since correlations were calculated using age-adjusted residuals, the effects specifically reflect the unique contribution of MSE to performance stability beyond maturation processes. Future research could explore multivariate models integrating multiple neural metrics to better understand the factors contributing to behavioral stability. These relatively small values of explained variance for individual differences add up with the much higher explanatory value of age for the relationships between MSE and behavioral variability.

## Figures and Tables

**Table 1:** Pearson correlation coefficients between age-adjusted residuals of behavioral variability measures at fine scales by region. *p*-values that remain significant after correction for multiple comparisons using the False Discovery Rate (FDR) method are indicated with asterisks.

Area		SD Oddball	CV Oddball
Left anterior	Pearson corr	−0.106	−0.132
	*p*	0.150	0.059
Middle anterior	Pearson corr	−0.099	−0.132
	*p*	0.150	0.059
Right anterior	Pearson corr	−0.082	−0.120
	*p*	0.217	0.068
Left central	Pearson corr	−0.120	−0.162 *
	*p*	0.150	0.041
Middle central	Pearson corr	−0.102	−0.128
	*p*	0.150	0.059
Right central	Pearson corr	−0.156	−0.188 *
	*p*	0.078	0.027
Left posterior	Pearson corr	−0.109	−0.137
	*p*	0.150	0.059
Middle posterior	Pearson corr	−0.128	−0.150
	*p*	0.150	0.052
Right posterior	Pearson corr	−0.163	−0.180 *
	*p*	0.078	0.027

SD: standard deviation, CV: coefficient of variation, corr: correlation.

## Data Availability

The data and code related to the present study are available upon reasonable request to the corresponding author (cgomez@us.es).

## Supplementary Materials

The following supporting information can be downloaded at https://www.mdpi.com/article/10.3390/e28040390/s1, Supplementary Table S1: Details of MSE Points, Sampling, and Frequencies.

## Abbreviations

The following abbreviations are used in this manuscript:

CV	Coefficient of Variation
DMTS	Delayed Match-to-Sample Test
EEG	Electroencephalography
FDR	False Discovery Rate
fMRI	Functional Magnetic Resonance Imaging
MSE	Multiscale Entropy
RTs	Reaction Time
SD	Standard Deviation
