# Supplementary material for: Age-Related Changes in EEG Signal Complexity and Behavioral Variability from Childhood to Adulthood: A Multiscale Entropy Approach
Source: Entropy (Basel). 2026 Apr 1;28(4):390. doi: 10.3390/e28040390 (PMC13115029; doi:10.3390/e28040390)
Supplement: Supplementary file 1 [file entropy-28-00390-s001.zip › entropy-4157496-supplementary.pdf]

SUPPLEMENTARY MATERIAL

Supplementary Table S1

A detailed description of the number of points. sampling periods. and frequencies associated with each MSE scale is presented. providing precise information on the temporal resolution and scale-specific parameters used in the analysis

Scales	Sampling period (ms)	Sampling frequency (Hz)	Number points trial	Nyquist Frequency	Lower Frequency
1	1.953	512	1024	256	0.5
2	3.906	256	512	128	0.5
3	5.859	170.67	341	85.33	0.5
4	7.813	128	256	64	0.5
5	9.766	102.40	204	51.20	0.5
6	11.719	85.33	170	42.67	0.5
7	13.672	73.14	146	36.57	0.5
8	15.625	64	128	32	0.5
9	17.578	56.89	113	28.44	0.5
10	19.531	51.20	102	25.60	0.5
11	21.484	46.55	93	23.27	0.5
12	23.438	42.67	85	21.33	0.5
13	25.391	39.38	78	19.69	0.5
14	27.344	36.57	73	18.29	0.5
15	29.297	34.13	68	17.07	0.5
16	31.250	32	64	16	0.5
17	33.203	30.12	60	15.06	0.5
18	35.156	28.44	56	14.22	0.5
19	37.109	26.95	53	13.47	0.5
20	39.063	25.60	51	12.80	0.5
21	41.016	24.38	48	12.19	0.5
22	42.969	23.27	46	11.64	0.5
23	44.922	22.26	44	11.13	0.5
24	46.875	21.33	42	10.67	0.5
25	48.828	20.48	40	10.24	0.5
26	50.781	19.69	39	9.85	0.5
27	52.734	18.96	37	9.48	0.5
28	54.688	18.29	36	9.14	0.5
29	56.641	17.66	35	8.83	0.5
30	58.594	17.07	34	8.53	0.5
31	60.547	16.52	33	8.26	0.5
32	62.500	16	32	8	0.5
33	64.453	15.52	31	7.76	0.5
34	66.406	15.06	30	7.53	0.5
